# Vulvar Apocrine Hidrocystoma: A Rare Case Report

**DOI:** 10.1155/crog/9794934

**Published:** 2026-09-17

**Authors:** Nastaran Mahmoudnejad, Peyman Mohammadi Torbati, Niloofar Irandoost

**Affiliations:** ^1^ Urology and Nephrology Research Center, School of Medicine, Shahid Beheshti University of Medical Sciences, Tehran, Iran, sbmu.ac.ir; ^2^ Pathology Department, School of Medicine, Shahid Beheshti University of Medical Sciences, Tehran, Iran, sbmu.ac.ir

**Keywords:** female, hidrocystoma, labia majora, vulva

## Abstract

Apocrine hidrocystoma is a rare and benign tumor that usually occurs on the head, neck, and face. Involvement of the vulva is extremely rare, with only a few documented case reports in the literature. The prognosis of the tumor is favorable with surgical excision, with no reported recurrences. Here, we present the case of a 40‐year‐old woman with a solitary nodule on the labia majora, without any other manifestations. We discuss its management and follow‐up course.

## 1. Introduction

Apocrine hidrocystoma (AH) is a benign and uncommon tumor with a cystic component that develops from the secretory section of the apocrine sweat glands. The tumor is typically found on the head and neck and presents as a single asymptomatic papule or nodule. It was first described by Robinson in 1893 [1]. AH can be translucent, with a typical size range of 3–15 mm, and is often mobile [2]. Whereas the benign tumor usually appears as a single growth, there are rare instances of multiple forms that may indicate specific hereditary disorders or ectodermal dysplasia, such as Schopf–Schulz–Passarge syndrome and Goltz–Gorlin syndrome. Histological evaluation is typically used to diagnose AH [3].

Sun exposure is considered a potential risk factor for AH, but it is not associated with a familial history of the disorder. The exact causes of AH are still unknown [2]. It rarely occurs in children or adolescents, with adults aged 30–70 being the most affected. Both men and women are equally likely to experience solitary AH, with no specific regional or ethnic preferences [4].

Dermoscopic evaluation reveals a nonconstant focal brownish‐orange area with linear vessels, as well as whitish cotton wool‐like features with a homogeneous pale gray or bluish pattern. Although AHs are clinically suspected, a histological evaluation is necessary for a definitive diagnosis [5–8]. Involvement of the vulvar area is extremely rare, with only a few documented case reports in the literature. The tumor should be considered a differential diagnosis of benign vulvar lesions. A definitive diagnosis will be made with an excisional biopsy and histopathologic examination. In this case report, we present a 40‐year‐old female patient with a well‐defined mass on her labia majora diagnosed as an AH. We will discuss the treatment, histopathologic findings, and follow‐up course of the patient.

## 2. Case Presentation

**Figure 1:**
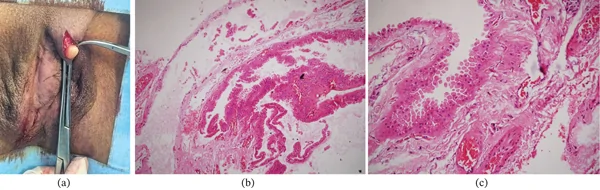
(a) Gross appearance of the lesion. (b) Unilocular cystic structure surrounded by a loose fibrotic capsule (H&E, 100x). (c) Bilayer epithelium including apocrine cells in the inner layer (H&E, 400x).

A 40‐year‐old woman was referred to our clinic with a complaint of a painless mass in her left labia majora. She mentioned that she had discovered the mass accidentally. The mass was mobile and painless. It gradually enlarged, prompting her to seek medical attention. During the physical examination, a solitary, mobile, nontender mass measuring 10 × 7 mm was found, with no other signs or symptoms present. There was no history of underlying disorders, familial, habitual, or drug use. The patient was deemed a candidate for mass removal. A surgical excision was performed on the left labia majora, and the mass was observed to be adhered to surrounding tissues (Figure 1a).

The mass tore during excision, resulting in the discharge of clear fluid. She was discharged from the hospital on the same day, and the postsurgical period was uneventful. During a 1‐year follow‐up, there were no related complications or recurrence of the mass. Histopathologic evaluation revealed a unilocular cystic structure along the subepithelial fibroconnective tissue. It was lined by double‐layered epithelium, consisting of an outer layer of flattened myoepithelial cells and an inner layer of tall columnar cells with ample eosinophilic cytoplasm and basally located round to oval vesicular nuclei. Apical decapitation in apocrine cells was visible, and there was also focal mild papillary projection (Figure 1b,c). These findings were consistent with AH.

## 3. Discussion

AH is an uncommon cystic tumor that is nonmalignant. It typically presents as a single dome‐shaped nodule, most commonly on the periorbital site. However, it can also appear on the scalp, neck, lips, ears, chest, and shoulders. In rare cases, it may even involve the genital areas in both genders [9, 10].

AH typically consists of an outer layer of flattened myoepithelial cells, which is similar to the secretory section of a normal apocrine gland, and an inner cyst wall lined with luminal columnar cells that indicate apocrine secretion. These cysts can present as either unilocular or multilocular. The epithelium is composed of a single or double layer of cuboidal–columnar cells located above the outer layer of myoepithelial cells. A bilaminar epithelium lines the cystic area, with columnar cells on the inner surface displaying eosinophilic traits and prominent luminal blebbing, known as apocrine snouts. Lipofuscin granules, as well as periodic acid–Schiff (PAS)–positive granules, are also present. Vascular connective tissue covered with secretory epithelium and papillary projections is also characteristic of AHs [11].

Based on our research, there have been few case reports regarding the occurrence of apocrine AH in the labia majora. We reviewed case reports from 2000 to the present. Chang et al., in 2001, reported a case of a 7‐year‐old girl with two black papules on her vulva. No other abnormalities were noted. Histopathological evaluation confirmed the diagnosis of AH [12]. In another report, Matsuyama et al. reported a 9‐year‐old girl with an AH on the genitalia without any other manifestations [13].

In two cases of AH mentioned above, similar to our case, the condition presents as a nontender nodule on the genitalia with no other signs or symptoms. Individuals are often only concerned about the appearance and nature of the nodule.

AH closely resembles many conditions and must be differentiated from cutaneous melanoma, basal cell carcinoma, blue nevi, follicular cysts, milia, syringoma, and eccrine cystadenoma.

Physicians should reassure patients about the benign nature of this lesion and inform them that treatment involves excision with narrow margins and histopathological evaluation [14, 15]. The primary focus of dermoscopy is to confirm the absence of features indicative of malignant tumors with similar clinical presentations, mainly basal cell carcinoma and amelanotic melanoma. However, the rare possibility of AH undergoing malignant transformation into hidradenocarcinoma should be considered, and patient follow‐up is advisable. The prognosis of the tumor is favorable with surgical excision, with no reported recurrences. Minimally invasive treatments like cyst puncture followed by trichloroacetic acid or hypertonic glucose injection have also been described [16, 17].

We believe that although the prevalence of AH in female genitalia is very rare, it should be considered a differential diagnosis for genital lesions. Complete surgical excision would be both diagnostic and therapeutic for the management of these lesions.

## 4. Conclusion

AH should be considered a differential diagnosis for benign lesions in the female genital area. Complete surgical excision can serve as both a therapeutic and diagnostic procedure. Recurrence of the lesion is rare.

## Author Contributions

Nastaran Mahmoudnejad: performing surgical procedure; Peyman Mohammadi Torbati: histopathological evaluation; Niloofar Irandoost: writing the manuscript.

## Funding

No funding was received for this manuscript.

## Ethics Statement

The patient gave her informed written consent and provided her medical records to the research team for the publication of the case report, with the approval and under the supervision of the University Ethics Committee and in accordance with the Declaration of Helsinki.

## Consent

Written informed consent to publish the data and photographs in medical journals was obtained from the patient.

## Conflicts of Interest

The authors declare no conflicts of interest.

## Data Availability

All data and medical records are available from the corresponding author on reasonable request.
